# Discontinuing statins or not in the elderly? Study protocol for a randomized controlled trial

**DOI:** 10.1186/s13063-020-04259-5

**Published:** 2020-04-19

**Authors:** Fabrice Bonnet, Antoine Bénard, Pierre Poulizac, Mélanie Afonso, Aline Maillard, Francesco Salvo, Driss Berdaï, Nathalie Salles, Nicolas Rousselot, Sébastien Marchi, Nathalie Hayes, Jean-Philippe Joseph

**Affiliations:** 1 CHU de Bordeaux, Service de Médecine Interne et Maladies Infectieuses, Saint-André Hospital, 1 rue Jean Burguet, F-33000 Bordeaux, France; 2 ISPED, INSERM U1219, Bordeaux Population Health Research Center, University of Bordeaux, F-33000 Bordeaux, France; 3 CHU de Bordeaux, Pôle de Santé Publique, Clinical Epidemiology Unit (USMR), F-33000 Bordeaux, France; 4 ISPED, INSERM U1219, Bordeaux Population Health Research Center, Team EMOS, UMR 1219, University of Bordeaux, F-33000 Bordeaux, France; 5 CHU de Bordeaux, Direction de la Recherche Clinique et de l’Innovation, F-33000 Bordeaux, France; 6 Département de Médecine Générale, University of Bordeaux, F-33000 Bordeaux, France; 7 ISPED, INSERM U1219, Bordeaux Population Health Research Center, Pharmaco-Epidemiology Team, UMR 1219, University of Bordeaux, F-33000 Bordeaux, France; 8 CHU de Bordeaux, Service de Pharmacologie Médicale, F-33000 Bordeaux, France; 9 CHU de Bordeaux, Service de Gériatrie, Hôpital Haut-Lévêque, F-33000 Bordeaux, France

**Keywords:** Primary prevention, Statins, Elderly patients, Cost-effectiveness analysis, Randomized controlled trial, Mortality

## Abstract

**Background:**

The risk/benefit ratio of using statins for primary prevention of cardiovascular (CV) events in elderly people has not been established. The main objectives of the present study are to assess the cost-effectiveness of statin cessation and to examine the non-inferiority of statin cessation in terms of mortality in patients aged 75 years and over, treated with statins for primary prevention of CV events.

**Methods:**

The “Statins in the elderly” (SITE) study is an ongoing 3-year follow-up, open-label comparative multi-centre, randomized clinical trial that is being conducted in two parallel groups in outpatient primary care offices. Participants meeting the following criteria are included: people aged 75 years and older being treated with statins as primary prevention for CV events, who provide informed consent. After randomization, patients in the statin-cessation strategy are instructed to withdraw their treatment. In the comparison strategy, patients continue their statin treatment at the usual dosage. The cost-effectiveness of the statin-cessation strategy compared to continuing statins will be estimated through the incremental cost per quality-adjusted life years (QALYs) gained at 36 months, from the perspective of the French healthcare system. Overall mortality will be the primary clinical endpoint. We assumed that the mortality rate at 3 years will be 15%. The sample size was computed to achieve 90% power in showing the non-inferiority of statin cessation, assuming a non-inferiority margin of 5% of the between-group difference in overall mortality. In total, the SITE study will include 2430 individuals.

**Discussion:**

There is some debate on the value of statins in people over 75 years old, especially for primary prevention of CV events, due to a lack of evidence of their efficacy in this population, potential compliance-related events, drug-drug interactions and side effects that could impair quality of life. Data from clinical trials guide the initiation of medication therapy for primary or secondary prevention of CV disease but do not define the timing, safety, or risks of discontinuing the agents. The SITE study is one of the first to examine whether treatment cessation is a cost-effective and a safe strategy in people of 75 years and over, formerly treated with statins.

**Trial registration:**

ClinicalTrials.gov: NCT02547883. Registered on 11 September 2015.

## Background

Cardiovascular (CV) events, including ischaemic heart disease and cerebrovascular diseases, are the leading cause of death worldwide and are a major cause of morbidity in industrialised and developing countries [1]. CV events are multifactorial, and their prevention is defined as a coordinated set of actions, at the population or individual level, intended to eliminate or minimise the impact of CV diseases and related disabilities.

According to the “2016 European Guidelines on Cardiovascular Disease Prevention in Clinical Practice”, total CV risk estimation based on a risk estimation system such as the Systematic Coronary Risk Evaluation (SCORE), which estimates the 10-year risk of fatal cardiovascular disease (CVD), is recommended for adults > 40 years old [2]. People are categorised as being at high risk (familial hypercholesterolaemia, blood pressure > 180/110, diabetes mellitus without organ damage, or moderate chronic kidney disease (CKD); glomerular filtration rate (GFR) 30–59 mL/min/1.73 m2)” and “calculated SCORE between 5% and 10%) or very high risk (documented CV disease, diabetes mellitus with target organ damage, severe CKD (GFR < 30 mL/min/1.73 m2), calculated SCORE ≥ 10%) for CV events”.

The 2019 American College of Cardiology (ACC) and American Heart Association (AHA) Guidelines on the Primary Prevention of Cardiovascular Disease also recommend that adults aged 40–75 years who are being evaluated for CV disease prevention undergo 10-year estimation of risk of atherosclerotic CVD [3].

Among the possible interventions to reduce CV risk, statins are recommended for secondary prevention in all patients with CVD and for primary prevention in patients with elevated low-density lipoprotein cholesterol (≥ 190 mg/dL) or with diabetes mellitus, and those determined to be at sufficient CV risk (high risk and very high risk) after a clinician–patient discussion of risk.

Statins are inhibitors of hydroxymethylglutaryl-CoA reductase and thus reduce the synthesis of cholesterol, especially low-density lipoprotein (LDL) cholesterol, an independent risk factor for CV events. Numerous large-scale randomized studies over the last 20 years have demonstrated the effectiveness of statins for reducing CV risk in primary and secondary prevention [4]. However, information on patients over 75 years old is lacking, as no randomized trials have been conducted in this population.

The European Guidelines and the 2019 ACC/AHA Guideline acknowledge that special considerations are required when prescribing lipid-lowering agents in older people because exposure to higher doses (or higher potency) may not increase life expectancy but may increase the risk of adverse effects. Assessment of risk status and a clinician–patient discussion of risk are needed to decide whether to initiate or continue statin treatment.

Statins represent one of the largest pharmaceutical industry markets in France; in 2014, around 5 million people were treated with statins, representing a total cost of 550 million euros. People 75 years and older represented 9.3% of the total population in 2019 [5]. We estimate that around 25% of these people are treated with statins, including around 40% in the context of primary prevention (target population over 500,000 people in France).

Given the absence of formal evidence of efficacy, and the high level of side effects, the risk/benefit ratio of primary prevention with statins in elderly patients has not been established. With no controlled studies to validate the benefits of statins in this population, there is some controversy in the literature about their use in this age group [6–16].

One previous trial implied that stopping statin medication therapy is safe and may be associated with a number of benefits, including improved quality of life (QOL) among patients with advanced life-limiting illness [17].

We postulate that statin cessation in patients older than 75 years, when prescribed for primary prevention of CV events, will be safe and may improve QOL through the absence of adverse effects, and lower the costs for the French National Health Insurance System.

The two main objectives of this study are therefore to assess the cost-effectiveness, in real-life use, of statin cessation in patients over 75 years old being treated for primary prevention of CV events and to examine the non-inferiority of statin cessation in terms of all-cause mortality in patients older than 75 years.

## Methods/design

### Trial design and settings

The Statins in the elderly (SITE) study is a 3-year follow-up, open-label, comparative multi-centre randomized clinical trial being conducted in two parallel groups in outpatient primary care offices. Our objective is to conduct a randomized controlled trial that is representative of usual care conditions. Our study is being conducted in general practices in France, and the eligibility criteria are limited, apart from regulatory requirements, to age and treatment with statins for the purpose of primary prevention.

All general practitioners (GPs) working in primary care centres in France can enrol patients in the study. They need only to use informatics devices to complete the electronic case report forms (e-CRF) during patient follow up.

### Eligibility criteria

The study population consists of patients aged 75 years and over who have no history of CV disease and who are being treated with a statin and regularly visiting their referent GP in France. Participants meeting the following criteria are eligible for inclusion: people aged 75 years and older who have been treated with statins for at least 1 year for primary prevention of CV events, and who give informed consent. Patients with hypertension and/or diabetes mellitus may be included.

Participants meeting any of the following criteria are excluded: progressive morbid disease and life expectancy of 3 months or less, diagnosed with dementia, suffering from known homozygous or double heterozygous familial hypercholesterolaemia, or unable to provide informed consent.

### Intervention

In the statin cessation strategy, the statins are stopped and patients are asked to end their treatment on the day of randomization. In the comparison strategy, patients continue their statin treatment at the usual dosage. Compliance with the use of statins is assessed by the Morisky questionnaire at each visit.

### Randomization

Randomization is being carried out centrally using the e-CRF on the day of the visit for participant inclusion, after the participant has signed the consent form. Participants are considered enrolled in the trial when randomized.

The randomization is unbalanced, with a 5:4 ratio in favour of the statin-continuation group to take into account a 20% risk that patients allocated to the statin continuation group will stop statins after randomization for any reason (side effects, patient’s decision, physician’s decision). The allocation sequence was elaborated using computer-generated random numbers, and was not stratified. To reduce predictability of a random sequence, we used blocks of varying size. A document describing the randomization procedure is kept confidential within the Clinical Epidemiology Unit.

Taking statins or discontinuing statins will not require alteration to usual care pathways (including use of any medication) and these will continue in both trial arms. Participants in the statin discontinuation group who experience a new vascular event will be allowed to restart statins at the discretion of the investigator and according to good clinical practice.

### Outcome measures

#### Economic endpoints

The cost-effectiveness of the statin cessation strategy compared to continuation of statins will be estimated based on the incremental cost per quality-adjusted life year (QALY) gained at 36 months, from the perspective of the French healthcare system. QALYs will be measured using the Euro-QOL five-dimension, three-level (EQ-5D-3 L) questionnaire at inclusion and at each follow-up visit [18]. This questionnaire has been validated in the French population.

Healthcare resources and costs will be extracted from the French Administrative Health Care Database (Système National des Données de Santé, SNDS) by a merging procedure, operated by the French National Health Insurance Fund and using data from the SITE trial. The SNDS gathers all reimbursement data for 98.8% of the French population [19]. The time horizon of the study (36 months) implies a discounting of costs and outcomes. In accordance with the methodological economic guidelines of the French National Health Authority (Haute Autorité de Santé, HAS), we will apply a 4% discount rate.

Following cost-effectiveness analysis (CEA), budget impact analysis (BIA) will provide useful information about the sustainability of the statin cessation strategy if it were applied to the entire French healthcare system. It will consider outpatient costs, hospital costs, medical and non-medical costs induced or avoided by stopping statins, and all budgetary implications related to changes in health management and/or health status improvement. The difference between induced and avoided costs will provide the net benefit to the healthcare system of widespread adoption of the statin cessation strategy for primary prevention among elderly individuals in France.

#### Main clinical endpoint

Overall mortality will be the primary clinical endpoint. As the investigators are GPs, they are informed of the death of their patients and will therefore constitute the source of mortality data in this trial.

#### Secondary endpoints

The secondary endpoints are:

- QOL score measured by the Quality of Life Scale SF12, which has been validated in older people
- Incidence of CV events
- Incidence of non-CV events (diabetes mellitus and cognitive disorders)

### Timeline and recruitment

This study employs the research network of French universities’ primary care departments (PCDs). Each PCD appoints a coordinating physician by geographic area to mobilise investigating physicians (486 expected in the study).

The recruitment of GPs all across France maximises representativeness and limits the impact of potential local practices. The representativeness of the investigators is being documented by collecting data about their sociodemographic profiles, activity, and training.

Patients are being recruited through consultations over a period of 36 months. Eligible patients not included in the study are being reported, along with the reason for non-inclusion.

### Conduct of research

Patients are approached and screened by GPs during their usual medical visit or on the renewal of their medication. Patients are first pre-included with their referent GP, and then after a time of reflection (that may be few minutes to days or months until the next visit), they sign the consent form with the GP and are randomized to one of the two strategies. On the consent form, participants are asked if they agree to use of their data should they choose to withdraw from the trial. Participants are also asked for permission for the research team to share relevant data with people from the Universities taking part in the research or from regulatory authorities, where relevant. This trial does not involve collecting biological specimens for storage. Table 1 outlines the different phases of the study and data collection.

**Table 1:** Design of the SITE study

Timepoint	Study period					
	Pre-inclusion T − 1	Inclusion T 0	M3 *+/−* 1 month	M12 +/− 3 months	M24 +/− 3 months	Close-out M36 +/− 3 months
**Enrolment**						
Information/eligibility screen	✓					
Standardised questionnaire^a^	✓		✓	✓	✓	✓
MMSE^b^	✓					✓
Consent signature		✓				
**Intervention**		✓	✓	✓	✓	✓
**Assessment**						
SF12 and EQ-5D-3 L Questionnaires^c^		✓	✓	✓	✓	✓
Clinical examination^d^	✓	✓	✓	✓	✓	✓
Biological tests^e^	✓(*)		✓	✓	✓	✓
ECG^f^		✓		✓	✓	✓
Clinical event data collection^g^		✓	✓	✓	✓	✓
Compliance^h^		✓	✓	✓	✓	✓
CVRF collection^i^		✓	✓	✓	✓	✓
SE/SAE collection ^j^		✓	✓	✓	✓	✓

^a^Standardised questionnaire with six questions to search for coronary disease history, stroke, or unnoticed peripheral artery disease

^b^Mini-Mental State Examination (French version, GRECO)

^c^Quality of Life Questionnaire SF12 (self-administered) and EQ-5D 3 L (self-administered)

^d^Clinical examination: examination as recommended by good clinical practices in the field of cardiovascular medicine (blood pressure, cardiovascular (CV) data, and lung auscultation)

^e^Laboratory tests: lipid (EAL), blood glucose, HbA1c (if diabetic), electrolytes, creatinine, creatinine clearance, and serum albumin. *In the absence of a balance sheet dated within 12 months before pre-inclusion, laboratory tests are prescribed at pre-inclusion and should be performed until the day of the inclusion visit

^f^ECG: electrocardiogram based on recommendations (once every 3 years if permanent arterial hypertension (HTA) and once a year if overt diabetes mellitus)

^g^Reports of significant clinical events occurring between visits

^h^Compliance assessed by Morisky questionnaire (eight items) at baseline (all patients) and during follow up (only in the group of patients in whom statin therapy is continued)

^i^Total CV risk factors: weight, waist circumference, smoking, hypertension, diabetes mellitus, previous family history of CV disease among first-degree relatives, high-density lipoprotein (HDL) and low-density lipoprotein (LDL) cholesterol

^j^SE/SAE: side effects and serious adverse events, respectively

### Sample size

According to data from the French National Institute for Statistics and Economic Studies (Institut National de la Statistique et des Études Économiques, INSEE) in the French population aged 75 years or older, we assume that mortality at 3 years in both study groups will be 15%.

The SITE sample size was computed to achieve 90% power in showing the non-inferiority of statin cessation in comparison to statin continuation, assuming a 2.5% unilateral *α* risk and a non-inferiority margin of 5% of the between-group difference in overall mortality at 3 years after randomization (nQuery Advisor software, v. 7.0). A 5% non-inferiority margin is fairly commonly reported in the literature, but 3-year follow up is not. This yields an annual non-inferiority margin of only 1.7%, which we think would be quite acceptable for the scientific community. We do not have to deal with multiple tests, as cost-effectiveness analyses do not require statistical testing [20].

We also assume that 20% of the individuals randomized to the statin-continuation group may spontaneously stop their treatment during follow up. Therefore, to ensure 90% statistical power in under-treatment analysis, it was decided to include 20% more individuals in the statin continuation group. In total, 2430 individuals will be enrolled in the SITE study, including 1080 in the statin cessation group and 1350 in the statin continuation group.

### Data management

The e-CRF is the primary data collection instrument for the study. Medical information for individual participants obtained as a result of this study is considered confidential, and disclosure to third parties is prohibited. The e-CRFs are labelled with a unique trial number.

Consent forms sent to Sponsors may contain patient identifiers for the purpose of monitoring, as described in the trial risk assessment. Such information will be stored in secure, locked storage. Monitoring is mainly performed through e-CRF and by telephone. However, on-site monitoring is performed after the 3-month follow up and after the last follow up in each of the recruitment sites. Auditing may be required by the Sponsor or by the French authorities (ANSM) and the process is independent from investigators and the Sponsor.

### Statistical methodology

In the non-inferiority analysis, the bilateral 95% confidence interval (CI) for the between-group difference in the 3-year mortality rate will be estimated. Considering < 5% change as clinically non-significant, we will consider non-inferiority demonstrated only if the 95% CI upper bound of the 3-year mortality difference between the two groups is < 5%. This analysis will primarily be performed according to an under-treatment principle. Formally, patients who do not receive the trial treatment as planned in the protocol will be excluded from the analysis. An intention-to-treat analysis will also be performed with the aim of maintaining the initial effect of randomization. The “missing = failure” strategy (failure being defined as death) will be used in the non-inferiority analysis.

Secondary clinical analyses will be conducted according to the intention-to-treat principle: all randomized patients will be analysed in the group to which they were initially randomized, and all of their data will be used regardless of eventual treatment changes during the study. As economic analysis is not based on statistical tests, no adjustment of the α-level is needed to deal with multiple comparisons. In all analyses, *p* < 0.05 will be taken to indicate statistical significance. A statistical team will analyse all data independently of the investigator.

The economic analysis will also be conducted according to the intention-to-treat principle. First, the point estimate of the incremental cost/utility ratio and its bootstrap 95% CI will be calculated. We will then estimate the incremental net monetary benefit (INMB) adjusted for potential confounding factors (adherence to statins at baseline, treatment duration, and particular statin dosage) using multiple linear regression models. Acceptability curves will then be plotted. The value of information will also be examined according to the principles proposed by Claxton [20].

### Safety and adverse event reporting

The adverse events expected during this research are mainly those related to the age of the study population, such as CV events, diabetes mellitus and/or its complications, cognitive disorders and dementia, falls, cancers, deaths, and adverse effects of concomitant therapies. Given that one group of patients will continue to use statins, adverse reactions listed in the summaries of product characteristics are also expected.

The Sponsor unit charged with security and vigilance must be informed immediately of any serious adverse events (SAEs), regardless of whether expected or unexpected, via the eCRF. This ensures continuous monitoring of safety of patients included throughout the trial. Moreover, annual safety evaluations will be conducted by the security and vigilance unit of the Sponsor, with particular focus on CV morbidity and all-cause mortality in both groups. The Data Safety Monitoring Board (DSMB) of the study will meet when 50% of the patients reach 12 and 24 months of follow up. We did not anticipate criteria by which the trial may be stopped, but the advice of the DSMB could be solicited in the case of unexpected safety issues or new information. In addition, all CV events will be validated by an external adjudication committee, which includes a cardiologist, a neurologist, and an internal and a geriatric medical specialist.

The Sponsor’s security and vigilance unit must communicate serious unexpected adverse reactions (SUSARs) and new information in the research in a timely manner in accordance with current regulations:

- To the French National Agency for the Safety of Medicines and Health Products, (Agence Nationale de Sécurité du Médicament et des Produits de Santé (ANSM)).
- To the research ethics committee (Comité de Protection des Personnes (CPP)). The committee will ensure, if necessary, that the research participants were informed about the side effects and confirm their consent.

On the anniversary date of first inclusion, the security and vigilance unit prepares an annual safety report including:

- A list of serious side effects of the research
- A concise and critical analysis of the safety of participants in the research

This report is sent to the ANSM and the CPP within 60 days of the anniversary date of first inclusion.

### Trial management

The scientific committee (SC) is composed of the coordinating investigator, Sponsor representatives, pharmacist, statistician, general practitioner, cardiologist, and geriatrics and experts in the field. The frequency of the SC meeting is at least once a year as needed during the research. The SC provides overall supervision of the trial; it will ensure that the study is carried out appropriately, scientifically, clinically and ethically. The SC is the decision-making executive body for all issues related to the trial implementation or follow up.

The DSMB is composed of six experienced external experts in the field of general practice, cardiology, geriatric medicine, methodology, and pharmacy, and are all nominated by the trial Sponsor. The DSMB is a consultative board for the SC and the Sponsor. It monitors the main safety and efficacy outcome measures and the overall conduct of the trial, with the aim of protecting the participants’ safety and interests. The DSMB will meet when 50% of the participants reach 12 and 24 months of follow up.

The day to day activities are supervised by a project coordination team that meets every 3 months and refers to the SC. The events validation committees will be in charge of validating neurovascular and cardiovascular events that occur during the course of the trial. This committee will meet to validate clinical events before the DSMB meeting to ensure that the results provided to the DSMB are consolidated.

### Trial registration

This research has been registered with ClinicalTrials.gov under the number NCT02547883.

### Amendment

To extend inclusions, the protocol was amended in March 2019 to also include patients from hospital departments.

## Discussion

The proportion of the population represented by people aged 75 years and older is increasing at a rapid rate in industrialised countries. According to the French National Institute of Demographic Studies (Institut National d’Études Démographiques, INED), life expectancy at age 75 years in France is 11.5 years in men and 14.4 years in women. Statins are among the most widely prescribed pharmacological treatments in this population.

Data from clinical trials guide the initiation of medication therapy for primary or secondary prevention of CV disease but do not clearly define the timing, safety, or risks of discontinuing treatment with these agents. As a result, the number of medications often accumulates, particularly in elderly people, with increased risk of drug–drug interactions and side effects.

There is debate on the value of statins in people aged 75 years and older, especially for primary prevention of CV events, particularly due to the lack of evidence of their efficacy in this population, potential compliance-related events, drug–drug interactions, and side effects that could impair the QOL of patients in this age group who are treated with statins [21–29]. In addition, the health economics literature does not demonstrate the cost-effectiveness of statin therapy for primary prevention in this population [30, 31].

To date, the SITE study is one of the largest studies conducted in France in the primary care setting among GPs, who are usually poorly trained with regard to randomized clinical trials. Therefore, to perform such a study, we have implemented a specific online questionnaire, which is as short as possible and includes only the key variables, to limit the problem of missing data, and have provided specific online courses to train the GP investigators in good clinical practices in the field of clinical research. We have also reinforced the data monitoring through our web system, the e-CRF, and phone calls made by experienced and trained research assistant. Also, we perform a systematic on-site monitoring visit after 3 months of follow up and after the last follow up at each of GP site. Finally, GPs are paid for their time spent on the trial (i.e. 50 euros per visit corresponding to two consultations).

Our study will have some limitations. First, we acknowledge that this is a pragmatic trial without blinding. The study participants and their physicians will know whether statin therapy has been continued or discontinued. The choice was to evaluate a strategy of discontinuing statins rather than to evaluate statin prescriptions alone. In theory, the absence of blinding could lead to a measurement bias due to greater surveillance of CV events in the discontinuation group. This will be closely monitored through the data from the SNDS. In addition, this is why the total mortality rate was chosen as our primary endpoint. Second, patients with previous dementia cannot be included in the trial, so our results will not be generalizable to this population. Third, although our sample is quite large, we cannot exclude a tendency from the investigators to recruit people with very few CV risk factors rather than patients with advanced disease and poor outcomes. Particularly, the inclusion of patients with diabetes mellitus will be monitored. Finally, patients treated with statins are being included in the SITE trial regardless of the specific type of statin. Therefore, we will not be able to examine specific treatments or dosages. However, we believe that the SITE study will be one of the first to show that a strategy of treatment cessation is cost-effective and does not adversely affect the prognosis of people over 75 years of age who are treated with statins.

## Trial status

Patient recruitment started on 15 June 2016. The expected recruitment completion date is 31 December 2019.

The protocol version number is 8.0, dated 5 June 2019.

## Data Availability

The datasets analysed during the current study are available from the corresponding author on reasonable request.

## Abbreviations

ACC: American College of Cardiology
AHA: American Heart Association
ANSM: Agence Nationale de Sécurité du Médicament
BIA: Budget impact analysis
CEA: Cost-effectiveness analysis
CI: Confidence interval
CPP: Comité de Protection des Personnes
CKD: Chronic kidney disease
CV: Cardiovascular
DSMB: Data Safety Monitoring Board
e-CRF: Electronic case report form
EQ-5D-3 L: Euro-QOL-5 Dimension-3 Level
GP: General practitioner
GFR: Glomerular filtration rate
HAS: Haute Autorité de Santé
LDL: Low density lipoprotein
INMB: Incremental net monetary benefit
INED: Institut national d’Etude Démographique
PCD: Primary Care Department
PRME: Programme de Recherche Medico-Economique
QALY: Quality-adjusted life years
QOL: Quality of life
SAE: Serious adverse event
SCORE: Systematic Coronary Risk Evaluation
SITE: Statins in the elderly
SNDS: Système National des Données de Santé
